# City comfort: weaker metabolic response to changes in ambient temperature in urban red squirrels

**DOI:** 10.1038/s41598-023-28624-x

**Published:** 2023-01-25

**Authors:** Bianca Wist, B. Karina Montero, Kathrin H. Dausmann

**Affiliations:** 1 Functional Ecology, Institute of Cell and Systems Biology of Animals, Universität Hamburg, Martin-Luther-King-Platz 3, 20146 Hamburg, Germany; 2 Biodiversity Research Institute (CSIC, Oviedo University, Principality of Asturias), Campus of Mieres, University of Oviedo, 33600 Mieres, Spain

**Keywords:** Ecophysiology, Urban ecology, Metabolism

## Abstract

The ecophysiological responses of species to urbanisation reveal important information regarding the processes of successful urban colonization and biodiversity patterns in urban landscapes. Investigating these responses will also help uncover whether synurban species are indeed urban ‘winners’. Yet we still lack basic knowledge about the physiological costs and overall energy budgets of most species living in urban habitats, especially for mammals. Within this context, we compared the energetic demands of Eurasian red squirrels (*Sciurus* *vulgaris*) from the core of an urban environment with those from a nearby forest. We measured oxygen consumption as a proxy for resting metabolic rate (RMR) of 20 wild individuals (13 urban, 7 forest), at naturally varying ambient temperature (T_a_) in an outdoor-enclosure experiment. We found that the variation in RMR was best explained by the interaction between T_a_ and habitat, with a significant difference between populations. Urban squirrels showed a shallower response of metabolic rate to decreasing T_a_ than woodland squirrels. We suggest that this is likely a consequence of urban heat island effects, as well as widespread supplemental food abundance. Our results indicate energy savings for urban squirrels at cooler temperatures, yet with possible increased costs at higher temperatures compared to their woodland conspecifics. Thus, the changed patterns of metabolic regulation in urban individuals might not necessarily represent an overall advantage for urban squirrels, especially in view of increasing temperatures globally.

## Introduction

Animals have to carefully balance their energy budgets in response to environmental conditions, such as fluctuations in ambient temperature (T_a_) or food availability^1^. The energetic demands of an animal in a certain environment are reflected by the metabolic rate (MR), which determines resource requirements and limits their allocation to body components, therefore directly influencing fitness^2,3^. Studying drivers of variation in MR is fundamental for understanding ecological patterns^2^. In general, MR shows high variability and phenotypic plasticity, both among and within species. Even among populations from different locations, which is assumed to reflect an adaptation to the particular habitat conditions^4–6^.

Urbanisation is a major driver of environmental change, and alongside the rapid loss of natural habitats poses a growing threat to biodiversity^7,8^. Urban areas have more than doubled from 1992 to 2015 with an even larger increase forecast for 2030, where 60% of the world’s population is projected to live in urban settlements^7,9^. Urban wildlife experiences pronounced differences in environmental conditions compared to geographically close rural populations, even though they are located at similar altitudes and latitudes^10–12^. Cities in particular are challenging habitats for wildlife due to immense human induced alterations and disturbances, such as noise, pollution or impervious surfaces^13,14^. However, some species, classified as synurban, seem to thrive in urban conditions displaying higher densities than in their natural habitats^15,16^. Urban populations often exhibit changes in biology and ecology^8,11,17^. For example, they show shifted and/or extended breeding seasons^16,18^, differences in body mass or condition^18–20^, and altered foraging and/or overall activity patterns^16,18,20,21^. It is likely that these changes are associated with altered physiological processes.

However, despite the central role of physiology, our understanding of its contribution to the adaptability of wildlife to urban conditions is still limited. Urban heat islands, characterized by higher T_a_, higher precipitation and altered wind velocity^22–24^ are assumed to alter or negatively affect the metabolism of urban wildlife^11,12,25,26^. Access to human-derived foods in urban habitats offer a more stable year-round food availability compared to rural or undisturbed natural environments^8^. As a consequence, animals in urban environments often differ in their seasonal fluctuations in body mass from their rural counterparts^20^. Furthermore, a diet that is supplemented by human foods and/or waste results in altered nutritional proportions, which can have knock-on effects on physiological and health-relevant traits^27–30^. Another factor that can influence MR are shifts in behaviour^1^. For example, exploratory behaviour is often elevated in urban settings^18^. Indeed, metabolism showed phenotypic correlation among populations of common voles (*Microtus arvalis*)^5^.

Here, we explored the role of physiological plasticity in enabling Eurasian red squirrels (*Sciurus* *vulgaris*, henceforth “squirrels”) to cope with urbanisation. Squirrels naturally occur in coniferous or mixed and deciduous forests, but are also highly abundant in urban habitat patches^16,31,32^. Their ability to successfully colonize urban environments makes them a valuable study system to disentangle drivers of synurbanisation with regard to metabolism. Despite being a small endotherm, this species does not use physiological energy saving strategies like torpor and remains homeothermic throughout the year^33,34^. This is remarkable, since small endothermic mammals are under strong pressure to maintain a careful balance between the costs of elevated body temperature and energy intake due to unfavourable surface area to volume ratios^35^. Previous work has demonstrated that squirrels from semi-urban environments show little seasonal variation in MR^34^ and instead, appear to rely mainly on behavioural adjustments like reduced activity during the winter^33,36–38^. As a food generalist and opportunist with a diverse diet, squirrels seem to benefit from urban food availability^32,39^. They mainly feed on seeds and nuts, but use a large variety of other food items when these are unavailable^37,40^. In urban areas, squirrels also feed on food sources provided by humans and exploit left-overs^39,41,42^. Akin with findings from other urban species^28,43^, the diet composition of urban squirrels can differ from their rural counterparts and they may feed on nutrient poor food items^44^. Furthermore, urban populations can be exposed to higher levels of intra- and interspecific contact rates, parasite transfer, stress, and exposure to environmental pollutants that might impact metabolism^11,25,45^.

To gain a better understanding of the physiological mechanisms driving synurbanisation, we compared resting metabolic rates (RMR) between wild-caught squirrels from the core area of a major city and squirrels inhabiting a nearby forest. We used a common garden approach, housing both populations in semi-natural outdoor enclosures, measuring MR with nest boxes as metabolic chambers to enable exposure to the same natural climatic fluctuations and to minimize experimental disturbances. We explored mass-specific RMR with regard to ambient temperature, habitat of origin and inter-individual variability. Additionally, to explore the influence of diel cycle, we compared RMR of squirrels during their active (day-time) and non-active (night-time) phases. The results of our study will advance the knowledge on physiological plasticity in the Eurasian red squirrel. Moreover, our work on urban ecophysiology contributes to the understanding of physiological demands and possible constraints or benefits for wildlife in highly urbanised habitats in general.

## Results

We captured 20 individuals (13 urban, 7 forest). Overall, we obtained a total of 57 measurement days (range of 1–5 per individual; n_forest_ = 22; n_urban_ = 35, Supplementary Table S2. Respirometry data and camera trap pictures showed that all squirrels left their nest box close to civil twilight to forage. The time spent outside the nesting boxes did not differ between urban and forest squirrels (Table 1, t-test: t = − 1.48, df = 18, *P* = 0.157). Forest squirrels were heavier than urban squirrels and had higher body condition indices (body mass (g)/nose-anus length (cm)), at the beginning (t-test, body mass: t = 3.60, df = 18, *P* = 0.002; BCI: t = 3.85, df = 18, *P* = 0.001) as well as after the experimental period (t-test, body mass: t = 3.37, df = 18, *P* = 0.003; BCI: t = 2.99, df = 18, *P* = 0.008). However, urban squirrels showed significant weight gain over the course of the experiment (paired t-test, t = − 2.53, df = 12, *P* = 0.027), whereas woodland individuals did not (see Table 1 and^44^ for further details). Urban individuals consumed on average 0.44 ± 0.13 ml O_2_ h^−1^ g^−1^ (0.33–0.52 ml O_2_ h^−1^ g^−1^) whereas RMR of forest squirrels was 0.48 ± 0.18 ml O_2_ h^−1^ g^−1^ (0.24–0.64 ml O_2_ h^−1^ g^−1^) over the measured temperature range of 8–29 °C. See Table 1 for a full overview of total and mass-dependent RMR results per group.

**Table 1:** Mean values (± SD) for body mass (before/after the housing period) and time spent outside the nesting box as well as mean values (± SD) and ranges (in square brackets) for mass-dependent and total RMR of forest (N_forest_ = 7, n_forest_ = 22) versus urban squirrels (N_urban_ = 13, n_urban_ = 35). Given are daily RMR averages (in ml O_2_ and in kJ), as well as diurnal and nocturnal values.

	Forest	Urban park
Body mass (g), before/after the experiment	371.4 ± 27.0/376.3 ± 19.5	334.2 ± 19.0/343.8 ± 21.1
Time spent outside the nesting box (h/measurement day)	3.1 ± 2.5	4.7 ± 2.1
RMR (ml O_2_ h^−1^ g^−1^)	0.48 (± 0.18) [0.24–0.64]	0.44 (± 0.13) [0.33–0.52]
RMR (ml O_2_ h^−1^)	173.52 (± 67.85) [84.50–232.09]	149.84 (± 44.40) [109.35–202.99]
RMR (kJ h^−1^ g^−1^)	0.0097 (± 0.0038) [0.005–0.013]	0.0091 (± 0.0027) [0.007–0.011]
RMR (kJ h^−1^)	3.54 (± 1.38) [1.72–4.73]	3.05 (± 0.90) [2.23–4.14]
Diurnal RMR (ml O_2_ h^−1^ g^−1^)	0.45 (± 0.18) [0.22–0.63]	0.42 (± 0.13) [0.30–0.50]
Diurnal RMR (ml O_2_ h^−1^)	164.34 (± 67.65) [79.83–229.36]	140.32 (± 41.72) [99.73–193.25]
Nocturnal RMR (ml O_2_ h^−1^ g^−1^)	0.53 (± 0.17) [0.26–0.68]	0.49 (± 0.13) [0.37–0.61]
Nocturnal RMR (ml O_2_ h^−1^)	194.67 (± 65.35) [95.33–278.72]	166.60 (± 44.05) [125.48–213.71]

**Figure 1:**
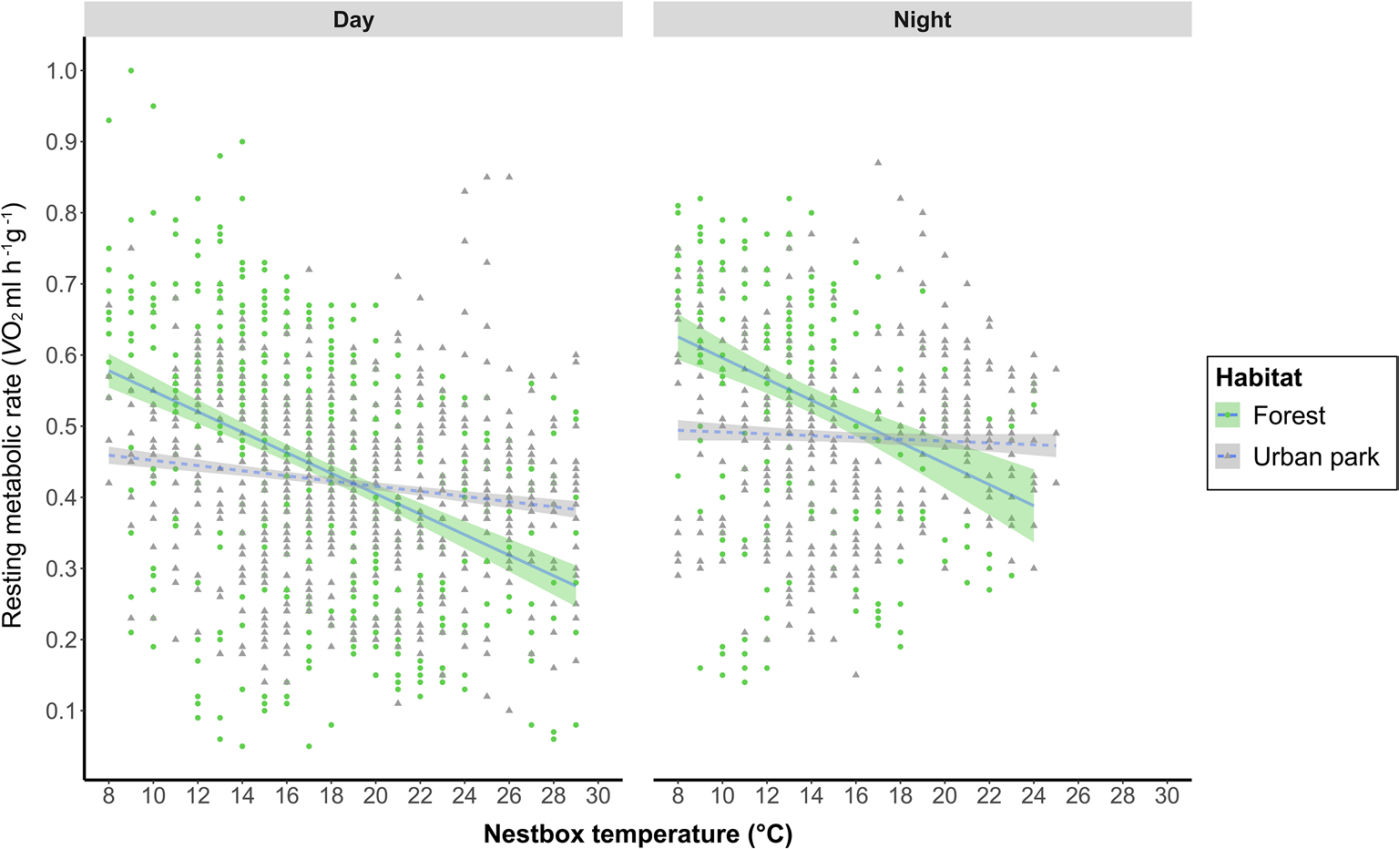
Fitted lines of model predictions and 95% CI band generated from the top ranked model as well as data points for diurnal (left panel) and nocturnal (right panel) mass specific resting metabolic rate (*V*O_2_ ml h^−1^ g^−1^) at measured ambient temperatures (diurnal range 8–29 °C, nocturnal range 8–25 °C) for forest (solid line, green band and green dots, N = 7) versus urban (dashed line, grey band and grey triangles, N = 13) squirrels (see Supplementary Figure S5 for graphs per individual).

We found statistical support for the interaction between nest box temperature (T_nest_) and habitat (ΔAIC_c_ = 4.79, Cohens *f*^*2*^ = 0.28, Tables 2 and 3), whereby RMR increased with decreasing T_nest_ in both groups (Fig. 1), but with a steeper slope in forest squirrels (Fig. 1). Furthermore, we found support for an effect of the phase in the diel cycle (ΔAIC_c_ = 5.79, Cohens *f*^*2*^ = 0.16, Tables 2 and 3). An effect of sampling period on RMR was marginally supported (ΔAIC_c_ = 2.02, Cohens *f*^*2*^ = − 0.27–0.13, Tables 2 and 3). Average RMR was higher during the night compared to daytime estimates (Fig. 1). We found no statistical support for an influence of the phase of the housing period (first/second half) nor the interaction of habitat and sampling period (Tables 2 and 3). RMR was highly variable across individuals (ICC 0.42, Table 3). The marginal R^2^ or conditional R^2^ respectively for the top ranked model was 0.304/0.597 (Table 3).

**Table 2:** The top ranked candidate linear mixed models evaluating the relationship between habitat type and covariates on RMR of Eurasian red squirrels.

Model no	Intercept	T_nest_	Habitat	D/N	Run	First/second	Habitat:run	Habitat:T_nest_	AICc	∆ AICc	W_i_(M)	ER
M1	0.610	− 0.0094	** + **	** + **	** + **			** + **	− 2941.849	0.000	0.488	
M2	0.612	− 0.0095	** + **	** + **	** + **	** + **		** + **	− 2939.834	2.015	0.178	2.738
M3	0.622	− 0.0095	** + **	** + **				** + **	− 2939.112	2.736	0.124	3.928
M4	0.570	− 0.0094	** + **	** + **	** + **		** + **	** + **	− 2937.938	3.911	0.069	7.068
M5	0.622	− 0.0096	** + **	** + **		** + **		** + **	− 2937.060	4.789	0.045	10.964
M6	0.550	− 0.0069		** + **	** + **				− 2936.062	5.787	0.027	18.054
M7	0.571	− 0.0094	** + **	** + **	** + **	** + **	** + **	** + **	− 2936.036	5.812	0.027	18.287
**Wi(V)**		**0.932**	**0.905**	**0.932**	**0.763**	**0.223**	**0.069**	**0.905**				

The models M1 to M6 sum up to a cumulative Akaike weight of 0.95 (= 95% confidence set). Parameters included in the model are indicated by “ + ”. AICc, Akaike’s information criterion, corrected for multiple parameters and small sample size; ΔAICc, differences in AICc; W_i_(M), Akaike weights per model, variate weights (W_i_(V)) for all predictors were calculated by summing up Akaike weights of the models containing the respective predictor; ER, evidence ratio; T_nest_, nest box temperature; habitat, habitat of origin; D/N, time of the day (day or night); run, sampling period (1–4), first/second, first or second half of the housing period.

**Table 3:** Coefficients for the predictors in the top ranked model (model no. 1, Table 2) complemented by standard errors (SE), confidence intervals (CI), degrees of freedom (df), standardized effect sizes (Cohen’s *f*^*2*^) as well as the intraclass correlation coefficient (ICC) and the marginal/conditional R^2^.

Predictors	Estimates	SE	CI	df	Cohen’s *f*^*2*^
(Intercept)	0.612	0.050	0.510; 0.714	26	0.000
Habitat (urban park)	− **0.103**	**0.048**	− **0.202; **− **0.005**	**32**	− **0.326**
T_nest_	− **0.010**	**0.001**	− **0.012; **− **0.007**	**1679**	− **0.320**
D/N (night)	**0.051**	**0.006**	**0.040; 0.062**	**1737**	**0.157**
Run (2)	0.050	0.051	− 0.058; 0.159	15	0.127
Run (3)	0.075	0.052	− 0.034; 0.185	16	0.223
Run (4)	− 0.091	0.055	− 0.208; 0.026	15	− 0.267
first_sec (first)	− 0.003	0.020	− 0.046; 0.039	17	− 0.011
Habitat(urban park):T_nest_	**0.005**	**0.002**	**0.002; 0.008**	**1643**	**0.279**
ICC	0.422				
Marginal R^2^/conditional R^2^	0.304/0.597				

Bold predictors indicate statistical significance with CI's not overlapping zero.

## Discussion

We used a common garden style experiment with semi-natural conditions to evaluate the energetic demands of Eurasian red squirrels from two contrasting habitats: an urban area and a nearby forest. Variations in energy expenditure were associated with T_nest_, however, the strength of this relationship was different depending on the habitat of origin of individuals. Among endothermic species, T_a_ represents one of the main factors influencing metabolism^46–49^ and several studies document physiological acclimatization to T_a_ over different seasons, latitudes or altitudes^4,50^. Generally, urban populations experience higher T_a_ (on average 0.5–3.0 °C) than their rural counterparts, especially at night^12,23^. This is also the case for the core areas of Hamburg where temperatures are on average up to 1.1 °C warmer than the surrounding areas, increasing up to 3 °C in summer^51^. As expected, energy expenditure in both of our experimental groups increased as T_nest_ decreased below the thermal neutral zone^34,52–54^. However, we did not find an overall lower RMR in urban individuals. Instead, we found that the magnitude of the effect of T_nest_, i.e. the increase in RMR per 1 °C change, was habitat-dependent.

We found that forest individuals showed a steeper slope of metabolic regulation, indicating a higher thermal sensitivity of RMR compared to urban squirrels. This supports previous findings of cold adaptation or temperature compensation in populations from colder climates, expressed by a higher RMR or a steeper relationship of RMR to T_a_^55^. Conversely, the apparently lower responsiveness of the urban squirrels indicates metabolic acclimatization to the warmer, local urban microclimate. Besides warmer T_a_, urban habitats display smaller diurnal urban T_a_ ranges^56,57^, which might have further contributed to the lower responsiveness in urban squirrel. Changes in MR driven by the thermal environment are usually closely linked to changes in thermal conductance, i.e., differences in insulation^58^. We assume that the urban squirrels differed in insulation-effective body components, e.g., in fur density or body fat compared to their forest counterparts. Seasonal changes in fur density have been documented in semi-urban squirrels^34^. Higher T_a_ combined with more stable resource availability leads to a buffering of seasonality in urban habitats^8^ and this might lead to different fur densities in urban vs forest squirrels. Interestingly, urban and rural bird nestlings differ in their number of feathers^59^ and similar insulation effective differences might occur in mammals.

Alterations in activity as found in several urban species including squirrels^18,60–62^ as well as changes in the diet could also lead to modifications in body composition, such as an increase in body fat and/or a decrease in muscle mass^63^, which may help explain our findings of different conductance between the groups. In urban areas, the scarcity of natural food items is often compensated by supplemental feeding^31,32,39^. We observed massive year-round supplementation in the urban habitat, but none in the forest (Wist et al., unpublished data). Surprisingly, body mass and condition were lower in the urban squirrels though. Despite higher food availability, urban wildlife often experiences low-quality diets, a shift in nutrient composition, or the ingestion of toxins and pollutants^20,28,30,43^. This can entail decreased digestibility of foods or lower processing efficiency, as well as other functional alterations, relevant for body mass and MR, such as changes in organ size (e.g., of the gut or liver), fat deposits or muscle mass^63–65^. Metabolic processes, such as efficiency in food digestion or in ATP generation, directly influence or even limit energy expenditure^1,66^. Moreover, diet quality and digestive efficiency together affect MR^67^. For instance, yellow bellied marmots (*Marmota flaviventris*), exhibit higher MR when fed a diet deficient in essential fatty acids^68^. The Talas tuco-tuco (*Ctenomys* *talarum*) was found to have lower MR when on a low-quality diet, in combination with a lower digestibility and higher gut transit^67^. South American foxes (*Pseudalopex* *cupaeus*) displayed increased MR when fed a mixed diet containing rats and fruits compared to a diet of only rats^69^. We found that urban squirrels chose food items higher in sugar content and more non-natural food items^44^. In combination with high supplementation, this could also have contributed to an increased insulation-effective fat layer in urban individuals and/or a different distribution of fat deposits between urban and forest squirrels, influencing conductance^63^.

Interestingly, as a result of the lower thermal sensitivity of RMR to changes in T_nest_, urban individuals spent less energy at the colder end of the T_nest_ range, whereas this pattern was reversed at higher T_nest_ (below/above ~ 19 °C during the day and ~ 18 °C at night). These findings contradict assumptions that higher urban temperatures are linked to a general increase in MR, e.g. by pollution induced metabolic costs^11^. However, this effect was mostly found in ectotherms and eco-physiological studies on urban mammals remain scarce. Notably, striped field mice (*Apodemus* *agrarius*) showed a reduced oxygen-carrying capacity in urban habitats, which was assumed to be caused by higher pollutant levels^70^. As the oxygen-carrying capacity limits the scope of MR^1^, this would also imply a decreased, rather than an increased MR in urban habitats. The comparably higher MR of urban squirrels at higher T_nest_ was an unexpected outcome. Endothermic mammals are not only challenged by the need for heat production at low T_a_, but by heat dissipation at high T_a_^52,71,72^. Despite possible shifts in thermal tolerance of urban wildlife^12,73^, urban mammals might be pushed towards their upper critical limits, suffering from over-heating and water loss, especially in the light of global warming. This could be further exacerbated by additional negative stressors such as urban noise or pollution^11,74,75^. Temperatures exceeding the above-mentioned threshold of ~ 18 °C for at least one hour occurred on 40% of the days in 2018—a comparably hot year and on 32% of the days in 2019 (Meteorological Institute, Universität Hamburg, Germany). However, urban squirrels might be less limited by heat dissipation, as they are less active and humans frequently provide year-round drinking water for birds and squirrels in surrounding gardens and on balconies (citizen survey data, Wist et al., unpublished). In addition, as urban squirrels respond less strongly to fluctuations in T_a_, the more stable rate of metabolism might be advantageous at the cellular level, as the body does not have to cope with pronounced, recurrent changes in homeostasis, which could possibly lead to e.g. cell stress, as is known from torpor-arousal-cycles^76^.

Independent of habitat of origin, phase in diel cycle was a relevant predictor of RMR in our model. As diurnal endotherms, squirrels usually display slightly lower body temperatures during the night^33,77^ and this is usually accompanied by a lower MR in an animal’s inactive phase^4^. Surprisingly, nocturnal RMR in our study was consistently higher in both urban and forest individuals than diurnal RMR at the same T_nest_. The drivers of this unexpected finding remain unclear. We assume that squirrels have to be able to elevate their metabolism radically as a prerequisite for quickly and suddenly climbing trees or jumping, e.g., in order to escape from predators. Diurnal periods of activity bursts might be followed by compensatory periods of extremely low RMR when resting. As we were only able to obtain MR data while the squirrels were in the nest boxes comparably low RMR values probably contributed disproportionately to the dataset although we statistically accounted for imbalances in the data. Moreover, the stress response in vertebrates seems to be highest during their inactive phase^78^, which might have contributed to elevated metabolism during the night in this study.

Besides the habitat-related differences, mass-specific RMR was highly variable across individuals. This is a well-known phenomenon, likely explained by diverse extrinsic and intrinsic factors, such as developmental conditions or genotype^45,79,80^. Historically, squirrels from both of our study sites had to adapt to similar environmental conditions due to their close geographic location. It is also plausible that gene flow occurred between them since an urban matrix does not always represent a barrier^81–83^. Nevertheless, there was still a clear effect of habitat on the thermoregulatory response of our two study groups. We are aware of the small sample size, particularly within the forest group and acknowledge that our results should be viewed with some caution. Our study also differs from many other studies in that we used a semi-natural set-up. Natural variability in environmental factors is often deliberately removed in physiological studies to reduce the effect of confounding variables. However, we aimed to expose the squirrels to as natural conditions as possible to express natural behaviour, using freshly caught individuals and largely undisturbed nest boxes with natural fluctuations in T_a_ and other climatic parameters to obtain biologically relevant results applicable to the field situation^84^.

## Conclusions

Our study gives valuable first insight into energetic demands of urban versus forest squirrels and thereby contributes to the understanding of ecophysiological consequences of urban heat islands on mammalian wildlife. This is particularly important in the light of rapid global urbanisation and climate change. Since resting metabolism sets limitations for resource intake and allocation to fitness components, physiological plasticity can be a key trait making squirrels successful colonizers in urban environments. However, we did not find an overall lower or higher RMR in the urban population, but more stable response to T_a_-fluctuations. The higher energy expenditures at higher T_a_ might indicate increased costs at temperatures that are expected to occur more frequently with the ongoing climate change^85^. There is an urgent need for studies exploring the interrelation of additional factors such as intrinsic processes related to diet or pollution with metabolism of urban mammals to provide a more comprehensive picture of the physiological consequences of urbanisation. Urban populations might be composed by “many losers and few winners”^86,87^ and we still do not know if synurban species thrive or rather persevere, even when occurring in high densities.

## Methods

### Trapping and handling

We trapped squirrels in a small park (Wohlerspark, 4.6 ha) located in the core city area of Hamburg, Germany (N53° 33′ 29.646" E9° 57′ 11.459") and in a forested site (Hahnheide, 1.450 ha) located approximately 30 km from the city centre (N 53° 37′ 14.146ʺ E10° 27′ 1.667ʺ). The park is characterized by a dense urban matrix of a city with 1.8 million inhabitants, resulting in very high human disturbance and various supplemental foods. The forest site is a nature reserve of mixed forest stands with many conifers and old trees. The mean year-round squirrel density in the park was much higher than in the forest site, (park = 5.1 squirrels/ha; forest = 0.1 squirrels/ha, minimum number alive, Wist et al., unpublished data). We used live traps (20 × 20 × 50 cm; Tomahawk Live Trap, Hazelhurst, Wisconsin, USA) with a seed and nut mix for bait. We opened the traps at 07:30 and checked them regularly until closing after 6–8 h. We used a cloth handling cone^88^ and individually marked captured animals using PIT-tags (ID-100B; Trovan Ltd., East Yorkshire, UK). We recorded body mass (spring-balance ± 5 g, KERN & SOHN GmbH, Balingen-Frommern, Germany), body length (nose-anus-length ± 0.5 cm, tape measure), sex, reproductive status and age (juvenile, subadult or adult, classified via body mass and reproductive status, following^89^). To minimize confounding effects on metabolism, such as sex or growth, we only selected adult males with no signs for disease. We transported squirrels to the Institute of Cell and Systems Biology of Animals, Universität Hamburg (53° 34′ 02.2" N 9 °58′ 45.6" E).

### Housing conditions

We held squirrels individually in large outdoor enclosures (average floor area 5 m^2^), under natural photoperiod, T_a_ and humidity. Housing facilities allowed us to house a maximum of six individuals at one time, resulting in four sampling periods (i.e. runs) that took place between the end of March and end of May during two consecutive years (2018/2019). We equipped each enclosure with branches to enable natural climbing behaviour and a nest box (Elmato 10,064 Großsittichkobel, Elmato GmbH, Holzheim, Germany, 30 × 22 × 20 cm, ~ 12 L), insulated with organic material (Pavatex, Pavaflex, Soprema GmbH, Germany). Individuals were housed between 13 and 18 days. Food (mix of foods usually encountered in their habitats) and water were offered ad libitum and changed every day at 2 p.m. (see^44^ for further details and Supplementary Figure S1 for a schematic sketch of the experimental set-up). We weighed all individuals again after the experiment to record possible changes in mass and released them back into their habitats at their capture sites.

### Ethical approval

All applicable institutional and national guidelines for the care and use of animals were followed. The authors complied with the ARRIVE guidelines. All procedures and animal handling were according to the German animal protection law and approved and authorized by the authorities of Hamburg and Schleswig–Holstein (general and housing permission by “Hamburger Behörde für Gesundheit und Verbraucherschutz”, permit no. 87/16, 17 November 2016 and permit no. 1/2018, 7 February 2018; exemption for the keeping of wild animals by the “Hamburger Behörde für Energie und Umwelt” and extension of the permits to Schleswig–Holstein by the “Ministerium für Energiewende, Landwirtschaft, Umwelt und ländliche Räume des Landes Schleswig–Holstein” (permit of 28 December 2016).

### Measurements of energy expenditure and temperature

We quantified RMR via oxygen consumption as ml O_2_ h^−1^ using open flow respirometry. Air from each animal was drawn directly from the nest boxes through airtight tubing (Tygon, Saint-Gobain, Paris, France). Oxygen content was quantified using portable oxygen analysers (OxBox 1–4, designed and constructed by T. Ruf & T. Paumann, FIWI, University of Veterinary Medicine Vienna, Austria), powered by a standard 12 V car battery^34,90–92^. By placing the measurement devices in a storage room next to the enclosures and using the nest boxes as respirometry chambers, disturbances to the animals were avoided since only the air tube already connected to the particular nest box had to be plugged into the oxygen analyser. We used a non-toxic modelling clay for sealing crevices and connections to minimise outflow contamination. Airflow was monitored by the flowmeter integrated in the set-up and set to 80 l/h. Oxygen content of the sample air was determined every ten seconds. As a reference, an hourly zero check was performed, i.e. oxygen content of the ambient air was analysed in the same interval for five minutes. We used silica gel to dry the air before entering the measurement devices. Calibration of the oxygen sensors was performed for each sampling period with calibration gas mixed by a gas mixing pump (Wösthoff Messtechnik GmbH, Bochum, Germany).

We measured RMR of the individuals after ~ 3 days of acclimation to the enclosures. We took measurements for periods of 24 h, starting in the early afternoon directly after food change, to enable recording of the complete inactive period at night as well as diurnal resting phases. Individuals were measured for a second 24 h period at the end of the housing period (day 12–17). Therefore, we obtained at least two measurement days for 16 out of 20 individuals to account for potential variation in MR throughout their time in captivity. Four individuals refused to use their nest box in the beginning, therefore, impeding the initial measurement. We measured a subset of individuals also in the middle of the housing period (see Supplementary Table S2). Ambient temperatures in the nest boxes (T_nest_) as well as T_a_ and humidity in the enclosures were measured in intervals of 10 min with loggers (Thermochron iButtons DS 1922/Hygrochron iButtons, DS1923L, resolution ± 0.5 °C, Maxim Integrated Products, San Jose, CA, USA). We also monitored some of the squirrels (n = 7) with camera traps (Snapshot Mini 5.0MP, DÖRR GmbH, Germany) to validate periods in which the individuals were outside and inside the nest box with the respirometry data.

### Data processing and statistics

We processed data-files from the oxygen analyser with Clampfit 10.3.1.4 (Molecular Devices, Sunnyvale, USA) to account for zero checks and exclusion of periods where the individuals were outside of the nest boxes (Supplementary Figure S6). We corrected measured values to standard temperatures and pressure and calculated the rate of oxygen consumption with the following equation^93^, which is applicable for our set-up^52,94^: ${V}^{^{\prime}}{O}_{2}=F{R}_{e}\frac{\left(Fi{O}^{2}-{F}^{^{\prime}}e{O}^{2}\right)}{\left[1-{F}_{i}{O}_{2}\left(1-RQ\right)\right]}$. FR_e_ represents the excurrent flow rate and F_i_O_2_–F′_e_O_2_ accounts for the difference in fractional O_2_ concentration when entering and leaving the nest box. We assumed a substrate utilization composed by 50% fat and 50% carbohydrates and thus used a respiratory quotient (RQ, ratio of CO_2_-production to O_2_-consumption) of 0.85^1,91,92,95^. We used the energy equivalence of 20.37 J/ml O_2_ to convert oxygen consumption into energy units^1,91^.

We included only the lowest 30% of RMR values per hour for analyses (RMR) to ensure excluding activity peaks^90^. Additionally, we categorized the data as day-time or night-time using civil twilight times. We then excluded the hour before and after official sunrise/sunset, since they represent potential transition periods from diurnal to nocturnal metabolism. To control for pseudo-replication, we then calculated RMR means per individual and hour of measurement day for T_nest_ rounded to the nearest integer. Clear upper outliers in T_nest_ (> 3 °C above T_a_), indicating an animal sitting close to or on the temperature logger were discarded. In those cases, we used the median of T_nest_ of the other nest boxes at the same time for the analysis. Due to the natural fluctuations of T_a_, and thus T_nest_, not all temperature integers were represented sufficiently frequently for robust analyses and were thus excluded. Therefore, we focused on RMR values for a temperature range between 8 and 29 °C. To obtain mass-specific RMR, we divided the values by individual body mass in g. We assumed a steady mass change from the start to the end of the experiment and used the estimated body mass for the particular day of measurement.

Data processing and statistics were performed in Excel (MS Office 2016) and R 4.1.2 (R Core Team 2021), respectively. We used the “RStudio” environment (RStudio Team 2021) and the packages “lubridate’^96^, “dplyr”^97^, “zoo”^98^ and “lattice”^99^ for initial data processing. We used linear mixed-effects models (LME) (lmer function within the package “lme4”^100^) and the package “lmertest”^101^ with RMR (ml oxygen consumption per hour and g of body mass; ml O_2_ h^−1^ g^−1^) as response variable. We tested all predictors for pair-wise correlations to avoid multicollinearity. We modelled T_nest_, habitat, phase in diel cycle (day/night), sampling period and time of housing period (first or second half of the housing period) as fixed factors in the full model. Graphical exploration (“ggplot2”^102^, “effects”^103^) revealed an interaction of habitat and T_nest_, as well as habitat and sampling period on RMR, therefore interactions were included in our model. We further allowed different intercepts and slopes per individual by including individual nested in phase of housing period as random factor to account for individual differences, repeated measurements across the individuals and the effect of the length of housing. See Supplementary Methods, Supplementary Table S3 and Supplementary Figure S4 for detailed information of the full model.

We used an information theoretic approach for our data analysis and performed model selection based on Akaike information criterion for small sample size (AIC_c_) and Akaike weights^104–106^. We further used the dredge function from the MuMIn package^107^ and model comparison with maximum likelihood. Normality and homoscedasticity were assessed by visual inspection of residual plots^108^. Since we included interactions in our models, model averaging was not feasible. Thus, we present the 95% confidence set of all possible models, i.e., models with sum of AIC weights ≤ 0.95^105,106^. To further obtain a measure of relative importance for each predictor, we summed up the AIC weights from all models of the confidence set containing the respective predictor^109,110^. Finally, we refitted the top ranked model with REML and report predictor estimates, confidence intervals (CIs) and local effect sizes (standardized coefficients or Cohen’s *f*^*2*^) for the predictors retained in the model^111,112^ as well as the marginal and conditional R^2^ (table created via “sjPlot”^113^). We interpreted estimates with CIs that do not overlap zero as evidence of model support and statistical significance^106^. Furthermore, we estimated the intra-class correlation coefficient (ICC) for the top ranked model as a measure of differences in RMR among individuals^114–116^. Unless otherwise stated, we present the data as mean ± 1 SD, N reports the number of individuals and n the number of measurement days.

## Supplementary Information

Supplementary Information.

## Data Availability

The datasets used and/or analysed during the current study are available from the corresponding author on reasonable request.
