# Supplementary material for: City comfort: weaker metabolic response to changes in ambient temperature in urban red squirrels
Source: Sci Rep. 2023 Jan 25;13:1393. doi: 10.1038/s41598-023-28624-x (PMC9876937; doi:10.1038/s41598-023-28624-x)
Supplement: Supplementary file 1 — Supplementary Information. [file 41598_2023_28624_MOESM1_ESM.pdf]

Supplementary material to:

City comfort – weaker metabolic response to changes in ambient temperature in urban red squirrels

Bianca Wist[1], B. Karina Montero[1,2], Kathrin H. Dausmann[1]

[1]Functional Ecology, Institute of Cell and Systems Biology of Animals, Universität Hamburg, Hamburg, Germany

[2]Biodiversity Research Institute (CSIC, Oviedo University, Principality of Asturias), Campus of Mieres, University of Oviedo, 33600 Mieres, Spain

Corresponding author: bianca.wist@uni-hamburg.de

S1

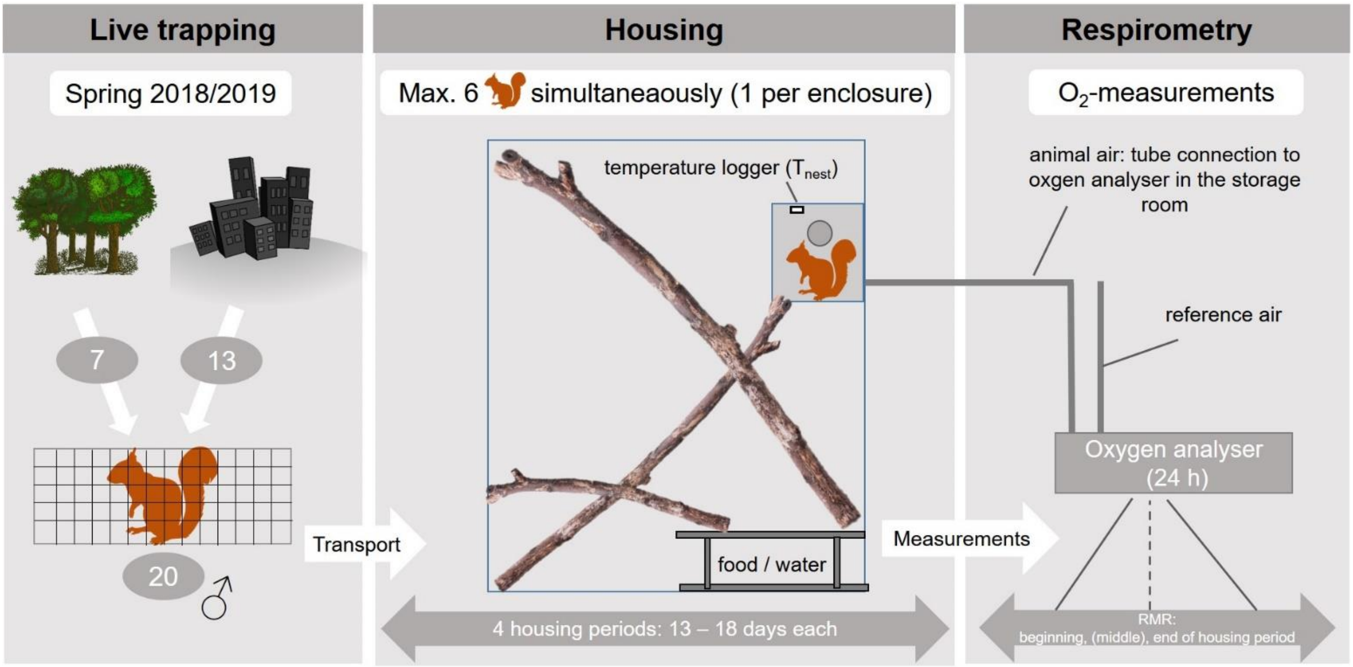

Supplementary Figure S1 Overview of experimental design: live trapping (left panel), housing conditions and length (middle panel) and respiromentry set-up (right panel)

S2

Supplementary Table S2: Overview of sampling effort per sampling period

Run	Year	Month	Individual no.	Habitat	Measurements (24 h)
1	2018	April	1	forest	2
			2	forest	1
			3	urban	2
			4	urban	2
			5	urban	2
			6	urban	2
2	2018	May	7	forest	2
			8	urban	2
			9	urban	2
			10	urban	3
			11	urban	3
3	2019	April	12	forest	5
			13	forest	3
			14	urban	1
			15	urban	4
			16	urban	3
4	2019	May	17	forest	5
			18	forest	4
			19	urban	4
			20	urban	5
**Total**			**20**		**57**

Supplementary Methods (statistical details)

Our full model was fitted with restricted maximum likelihood (REML) and described by the following structure (Supplementary Tab. 2):

ml O_2 h^{-1} g^{-1} ~ habitat + T_{nest} + D/N + run + first_sec + habitat:run + habitat:T_{nest} + (1+first_sec | individual), data = eich, REML = T

S3

Supplementary Table S3: Overview of predictors included in the full model

Variable	Type of predictor	Abbreviation
Nest box temperature	fixed effect, continuous	T_{nest}
Habitat of origin (forest/urban park)	fixed effect, factor with 2 levels	habitat
Phase in diel cycle (day/night)	fixed effect, factor with 2 levels	D/N
Time of housing period (first/second half)	fixed effect, factor with 2 levels	first_sec
Sampling period	fixed effect, factor with 4 levels	run
Habitat and sampling period	fixed effect, interaction	habitat:run
Habitat and nest box temperature	fixed effect, interaction	habitat:T_{nest}
Individual nested in first/second half of the housing period	random effect, nested	first_sec|individual

S4

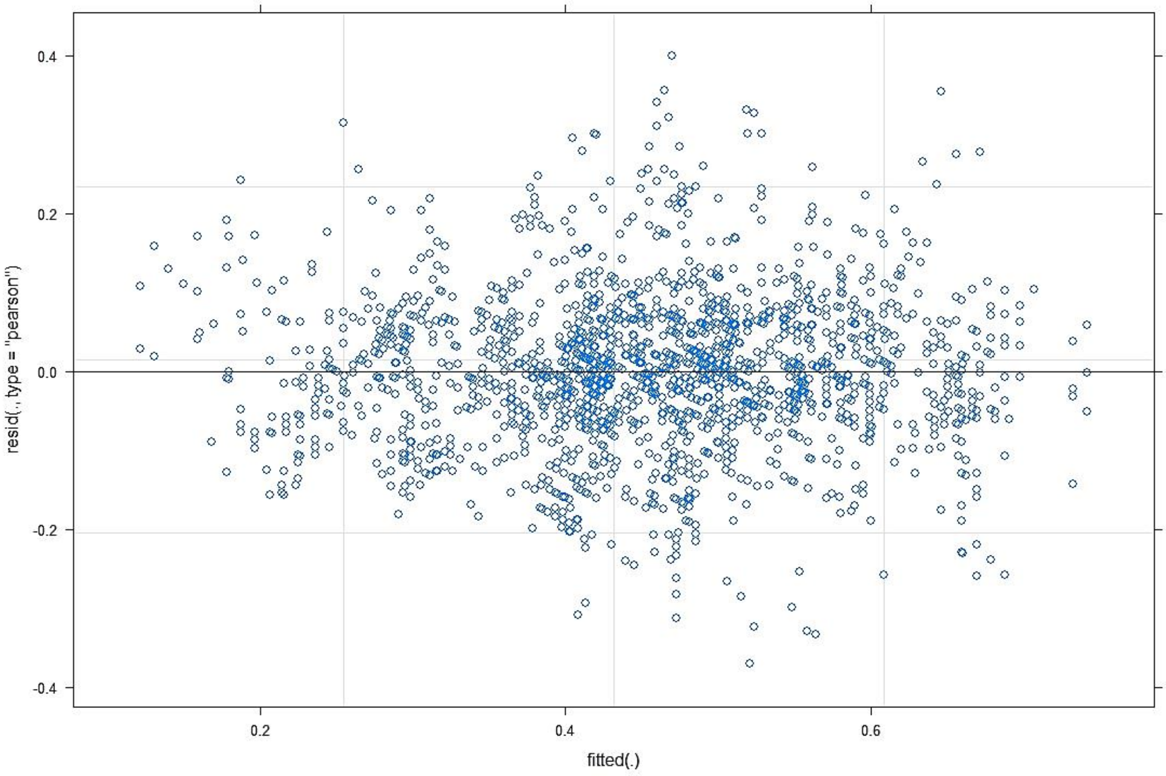

Supplementary Figure S4 Diagnostic plot with fitted values versus residuals created from the full model.

S5

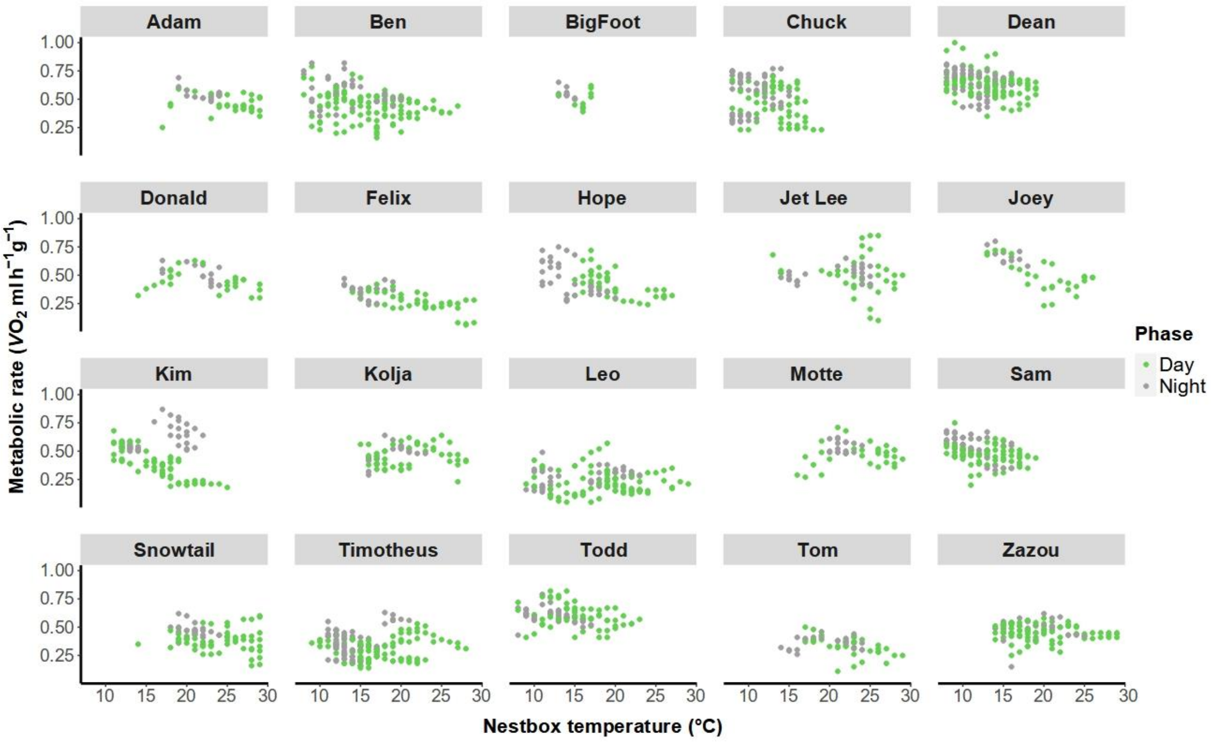

Supplementary Figure S5 Data points from all measurement days per individual for diurnal (green dots) and nocturnal (grey dots) mass specific metabolic rate (*V*O^2 ml h^{-1} g^{-1}) at measured ambient temperatures (diurnal range 8 – 29°C, nocturnal range 8 – 25°C).

S6

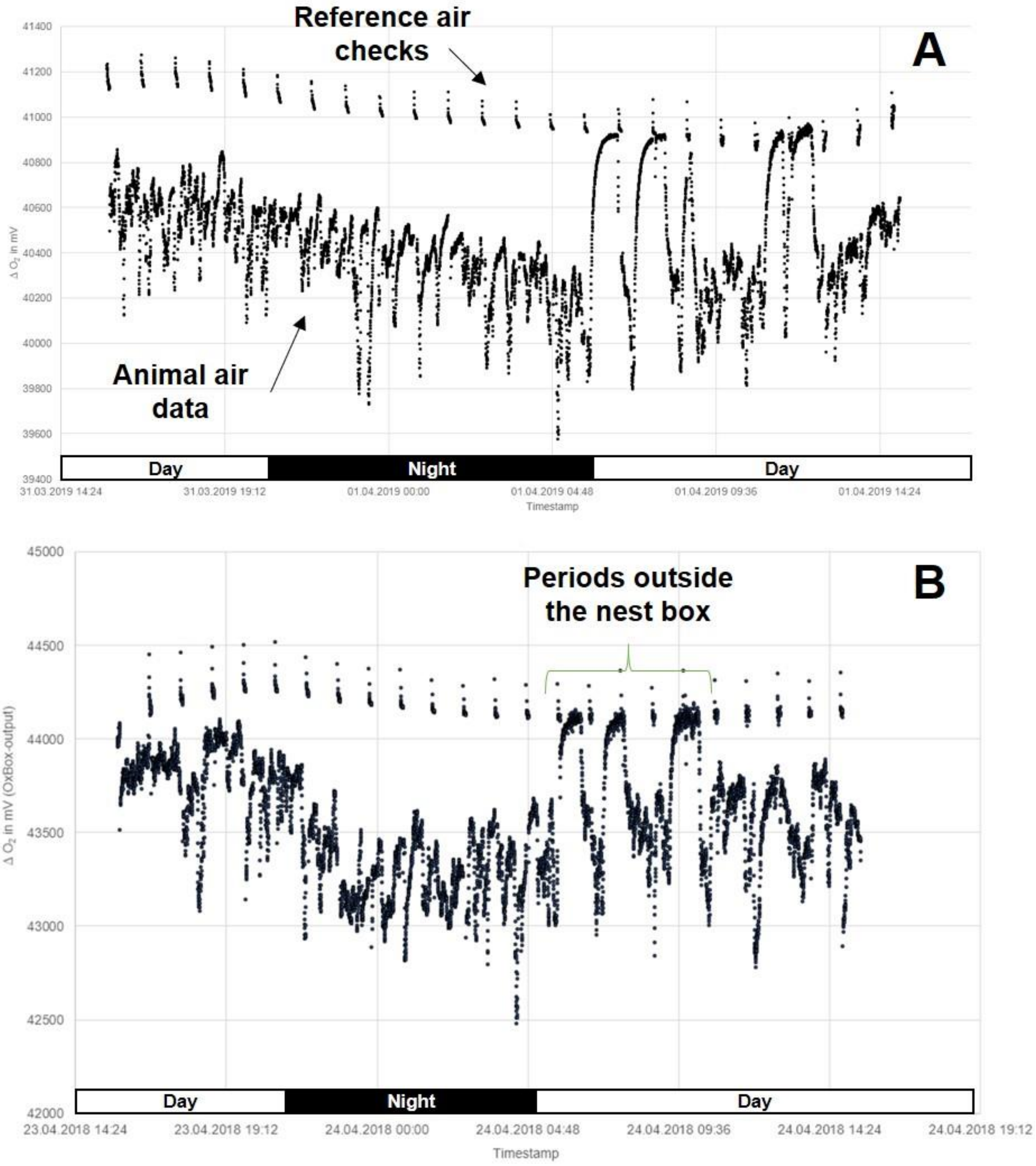

Supplementary Figure S6 Two examples of the raw data output from the urban individuals Sam (A) and Kim (B). Difference in oxygen content of the animal air/reference air in mV are plotted over time. The measurement curve includes hourly reference air checks as well as periods where the animals left their nest boxes (usually around sunrise to forage). In these cases, the data points generated from the animal rapidly dropped to the level of reference air checks (ambient air). These timespans were discarded prior to analysis. Day and night are shown by the white or black bars and correspond to local civil twilight data. Higher nocturnal values were already detectable within these raw data outputs (indicated by an average higher distance to the reference air checks). Double checking these outputs was part of our steps to validate the data and the unexpected outcome of a higher nocturnal RMR.
